# Mushroom biomass and diversity are driven by different spatio-temporal scales along Mediterranean elevation gradients

**DOI:** 10.1038/srep45824

**Published:** 2017-04-06

**Authors:** Josu G. Alday, Juan Martínez de Aragón, Sergio de-Miguel, José Antonio Bonet

**Affiliations:** 1 Departament de Producció Vegetal i Ciència Forestal, Universitat de Lleida-Agrotecnio Center (UdL-Agrotecnio), Avda. Rovira Roure, 191, E-25198 Lleida, Spain; 2 Centre Tecnològic Forestal de Catalunya (CTFC-CEMFOR), Ctra. de St. Llorenç de Morunys km 2, E-25280 Solsona, Spain; 3 Forest Bioengineering Solutions S.A. Crta. de St. Llorenç de Morunys, Km. 2, E-25280 Solsona, Spain

## Abstract

Mushrooms are important non-wood-forest-products in many Mediterranean ecosystems, being highly vulnerable to climate change. However, the ecological scales of variation of mushroom productivity and diversity, and climate dependence has been usually overlooked due to a lack of available data. We determined the spatio-temporal variability of epigeous sporocarps and the climatic factors driving their fruiting to plan future sustainable management of wild mushrooms production. We collected fruiting bodies in *Pinus sylvestris* stands along an elevation gradient for 8 consecutive years. Overall, sporocarp biomass was mainly dependent on inter-annual variations, whereas richness was more spatial-scale dependent. Elevation was not significant, but there were clear elevational differences in biomass and richness patterns between ectomycorrhizal and saprotrophic guilds. The main driver of variation was late-summer-early-autumn precipitation. Thus, different scale processes (inter-annual vs. spatial-scale) drive sporocarp biomass and diversity patterns; temporal effects for biomass and ectomycorrhizal fungi vs. spatial scale for diversity and saprotrophic fungi. The significant role of precipitation across fungal guilds and spatio-temporal scales indicates that it is a limiting resource controlling sporocarp production and diversity in Mediterranean regions. The high spatial and temporal variability of mushrooms emphasize the need for long-term datasets of multiple spatial points to effectively characterize fungal fruiting patterns.

The study of productivity and biodiversity changes along elevational gradients is an important theme in ecology and environmental management^1^. Elevational gradients provide invaluable information about the role of a changing climate on terrestrial biomass and biodiversity, providing fundamental information to assist in anticipating possible changes in ecosystem structuring and functioning brought about through global warming. General circulation models consistently indicate that climate change will be significant in the Mediterranean biogeographic region, lending increased urgency for the need to describe and understand climate interactions between terrestrial organisms^2^. Recent studies have provided evidence of climate-induced elevational shifts in both vegetation^3^,^4^, and fungal productivity and diversity^5^,^6^,^7^,^8^. The importance of forest fungi, besides being symbionts and decomposers^9^, is based on their potential to migrate rapidly and hence colonize new areas faster than some tree or shrub species^10^,^11^. As a consequence, movement of fungi and their fruiting bodies could be used as proxies to monitor early climate change impacts on forest communities, especially in sensitive areas such as the Mediterranean biogeographic region.

Fungi have significant effects on ecosystem processes and services at a range of scales, ranging from local to global. Hence, climate-induced changes in phenology and productivity of fungi fruit bodies (i.e. sporocarps^12^,^13^), which allow for sexual reproduction and enable long-distance spore dispersal^14^,^15^, can have wide-ranging consequences. For instance, ectomycorrhizal fungi in symbiosis with living tree roots directly influence tree nutrient, water availability^16^ and system productivity, while saprotrophic species influence decomposition^17^, being fundamental in soil and above-ground carbon storage on soil-plant systems^18^. Thus, at the global scale, the effects of future warming on forest ecosystems may depend to some extent on (i) whether fungi are resistant or not to climate-induced changes, and (ii) whether fungi are able to colonize new areas, which means that their reproductive activity should be sustained in time guaranteeing a spore flow^19^. At the local scale, reductions in fungal species richness and productivity are expected to reduce ecosystem biodiversity and services (regulating and provisioning), with strong ramifications on local livelihoods such as mushroom-picking^6^,^20^.

There is ample reason to expect that there will be variations in spatial and temporal responses of mushrooms to climate warming^21^. If this were so, we should expect important shifts in mushroom distributional ranges in areas where climate warming is greater, for example in Mediterranean areas^5^,^22^. Understanding the temporal variability of epigeous sporocarps along elevational gradients (climatic gradients) and the factors driving variability provides initial information to plan for future sustainable management of wild mushroom production in Mediterranean areas^20^.

Few studies have investigated how mushroom abundance and diversity vary along elevation gradients in Mediterranean areas, with most studies being done either in temperate\boreal^23^,^24^ or tropical zones^25^. Therefore, analysing these effects in Mediterranean areas could provide additional insights into fungal responses to climate change. Elevational gradients in localised areas provide “natural” experimental setups to test abundance and diversity variability produced by global warming. Recent studies have demonstrated the importance of temperature, precipitation and their interactions as drivers of ectomycorrhizal and saprotrophic mushroom production and diversity dynamics^26^. However, it is unclear if both fungal groups display similar changes in fruiting pattern in response to elevation and climatic drivers simultaneously.

During the last century both temperature and precipitation regimes have changed worldwide. Moreover, by 2085 in Mediterranean European mountains it is predicted that temperatures increase by 1.6° to 8 °C and precipitation decrease by 20%^27^. However, the idea that fungal productivity and diversity may shift towards elevations that offer an optimal set of climatic conditions remains to be tested. Clearly, rapid changes in climate are predicted to produce the most dramatic effects at species range-edges^28^. As a consequence, sporocarp productivity might be altered rapidly in sub-optimal areas before range shifts occur^20^,^29^; e.g. at lower elevations where temperatures increases and precipitation decreases will most likely accelerate aridification and reduce fungi sporocarp productivity^6^. These fungal shifts might have a short/medium-term effects on total yield and associated provisioning of forest ecosystem services.

Previous surveys have documented contrasting results regarding sporocarp diversity responses along elevational gradients. For example, Nouhra *et al*.^23^ found that sporocarp richness and biomass were correlated negatively with elevation in *Nothofagus* stands in Argentina. However, ectomycorrhizal fungal richness patterns in Japan showed a mid-elevation peak^30^, which is consistent with the mid-domain effect^31^. In contrast, in the western Mediterranean basin, we would expect sporocarp richness and biomass upward shifts in elevation in response to warmer and drier scenarios at the lower elevational edge. These contrasting observations emphasize the need to determine the implications of the rise in global temperatures recorded during the last decades on sporocarp diversity and productivity in different biogeographical regions.

In this paper, we tested whether diversity and productivity of epigeous fungal sporocarps changed in Scots pine (*Pinus sylvestris*) stands growing in a Mediterranean climate over and eight-year period by measuring biomass and diversity patterns along an elevational gradient (684 to 1615 m a.s.l.). Given that Scots pine in Spain reaches the most arid limit of its distribution^32^, we expect that environmental variations across a wide elevation gradient might result in sporocarp biomass and diversity differences, particularly at elevations that are far from the optimal growing conditions of Scots pine and its associated fungi. Additionally, we investigated the unknown relationship of epigeous sporocarp evenness with elevation and time. Evenness provides information of the equitability of fungal sporocarp species abundances in a given community, being an important factor to fully understand the reproductive structure and function of fungal communities.

In line with these premises, we tested two main hypotheses: Hypothesis 1: epigeous sporocarp biomass and diversity will vary through time along the elevation gradient; there are three sub-hypotheses: (i) temporal variation in sporocarp biomass and diversity will be greater that spatial variation (i.e. weather differences between years more important than differences between localities and plots), (ii) mycorrhizal and saprotrophic functional guilds will respond differently at the different ecological scales^20^, and (iii) fluctuations will be greater in the lower and upper extremes of the elevational gradient, i.e. more distant from fungal community optimal conditions. Hypothesis 2: elevation will affect sporocarp biomass and diversity, with three conflicting sub-hypotheses for species richness: (i) sporocarp richness will decline with elevation^23^, (ii) sporocarp richness will produce a peak at middle elevations^30^ and (iii) sporocarp richness will increase with elevation.

## Results

We recorded 41,588 epigeous sporocarps from at least 580 species in 117 genera and 51 families between 2007 and 2014. The fungal mushroom community abundance, productivity and richness were dominated by mycorrhizal species with 27,546 epigeous sporocarps, representing at least 349 different species, and accounting for 94% of the total collected biomass. Saprotrophic accounted for 14,042 epigeous sporocarps of at least 231 different species and representing the 6% of the biomass. The list of the 100 most productive species can be found in Supplementary Table S1.

### Ecological scales of variation in sporocarps

**Figure 1:**
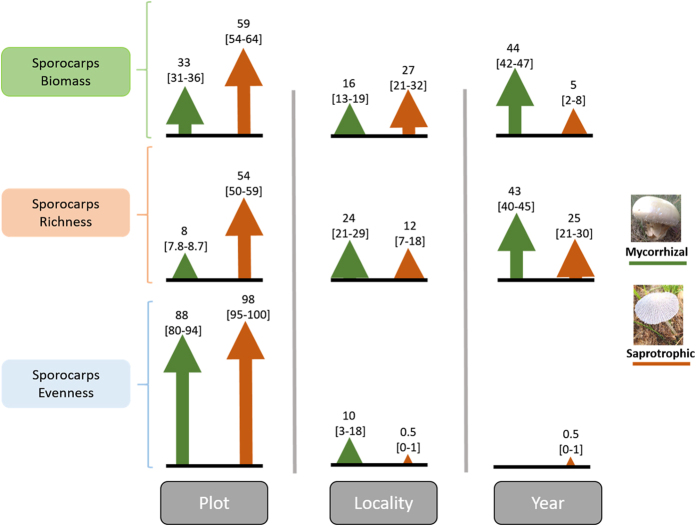
Arrows indicating the percentage of variance for biomass and diversity variables across three ecological scales (plot, locality and year) for mycorrhizal and saprotrophic functional guilds. Bootstrapped 95% confidence intervals are represented inside square brackets.

The partitioning of variance for biomass showed a greater distribution of variance across years (42%), followed by plots (34%) and localities (17%, Table 1). Interestingly, the two functional guilds revealed clear differences at the scale at which most of the biomass variation occurs; years accounted for 44% of total variance in biomass for mycorrhizal fungi, whereas plots accounted for 59% for saprotrophic fungi (Fig. 1). The variance partitioning for richness showed a fairly balanced variance distribution across the three scales considered: plots (35%), localities (29%) and years (28%, Table 1). However, considering the functional guilds separately again there were clear differences at the scale at which most of the variation occurs; years accounted for 43% of total variance for mycorrhizal fungi richness, whereas plots accounted for 54% of total variance for saprotrophic fungi richness (Fig. 1).

In contrast, variance partitioning of evenness showed a completely different variance distribution among ecological scales when considering biomass and richness variables (Table 1). Here, most variation in evenness occurs between plots (86–98%), with residual values for years and localities (<10%, Fig. 1). At the same time, the additive modelling demonstrated that there was no significant temporal trend in any evenness variables (total, mycorrhizal and saprotrophic; *P* > 0.05, Table 2), indicating that the three evenness variables had similar values. Total epigeous sporocarps mean evenness was 0.61 ± 0.02 (±SE; range 0.19–0.93); values for mycorrhizal evenness was 0.64 ± 0.02 (range 0.11–0.94) and saprotrophic evenness was 0.60 ± 0.02 (range 0.10–0.99).

### Sporocarp biomass through time and elevation

**Figure 2:**
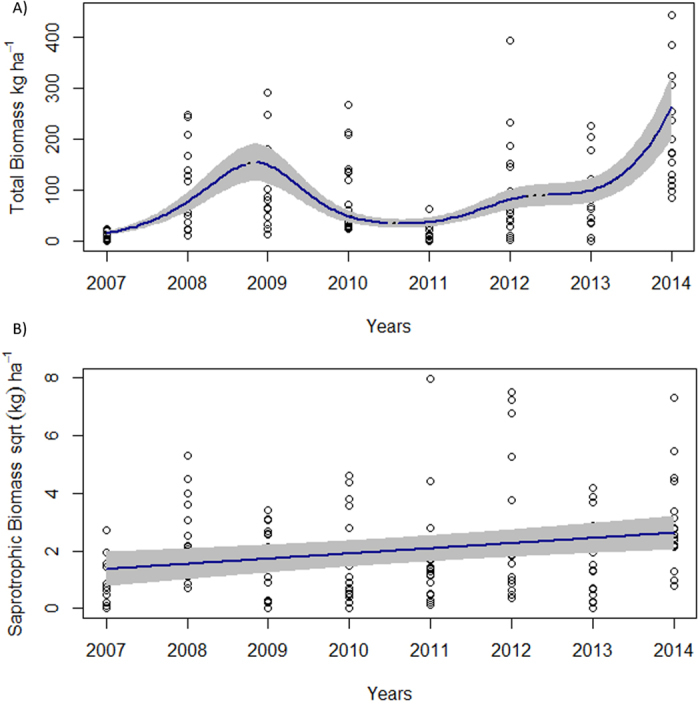
Temporal patterns fits of epigeous sporocarps total annual biomass (kg ha^−1^) in *P. sylvestris* forest stands along an elevational gradient within a Mediterranean region (Catalonia): (**A**) total biomass, (**B**) square-root of saprotrophic biomass. Each point represent a sample plot. Continuous blue lines indicate significant temporal decreases or increases in richness (*P* < 0.05; GAMMs). Grey envelops are 95% confidence intervals.

Temporal variation of epigeous sporocarp biomass was non-linear for total and mycorrhizal fungi, but saprotrophic biomass showed a significant increasing linear trend through time (Fig. 2). As mycorrhizal species represented 94% of total biomass both trends through time were almost identical and were characterized by a significant biomass increase from 2007 to 2009 up to 133 ± 22 kg ha^−1^ and 129 ± 21 kg ha^−1^, respectively (hump-shape; Fig. 2A). Then, there was a significant reduction until 2011 (total = 25 ± 10 kg ha^−1^; mycorrhizal = 17 ± 8 kg ha^−1^), followed by a significant increase until 2014 when a production maximum is reached (total = 255 ± 35 kg ha^−1^; mycorrhizal = 241 ± 34 kg ha^−1^). In contrast, the saprotrophic biomass showed a slightly linear increase from 2 ± 1 to 6 ± 2 kg ha^−1^ through the eight-year period (2007–2014, Fig. 2B).

The three biomass variables analysed (total, mycorrhizal and saprotrophic) showed no significant trends with elevation (*P* > 0.05; Table 2). However, analysis of elevational trends by years revealed two significant opposing linear trends for total, mycorrhizal and saprotrophic biomass; a decreasing trend in 2012 (slope_total_ = −2.19 ± 0.95, *P* = 0.023; slope_sapro_ = −0.89 ± 0.35, *P* = 0.013) and an increasing trend in 2013 (slope_total_ = 3.48 ± 0.95, *P* *<* 0.001; slope_sapro_ = 0.90 ± 0.35, *P* = 0.011). The climatic analysis showed that the most important driver for the three epigeous biomass variables was LSEA precipitation (greater fixed parameter estimates than temperature: 6 vs. 1.5; Table 2). Total, mycorrhizal and saprotrophic biomass showed positive significant relationships with precipitation (*P* < 0.05; Table 2), indicating that sporocarp biomass increases with increasing LSEA precipitation (positive slope), regardless of the functional guild considered. However, LSEA mean temperature was only significantly positively related with total and mycorrhizal biomass (*P* < 0.05; Table 2), indicating a slightly increase in biomass with increasing LSEA mean temperature.

### Sporocarp diversity through time and elevation

**Figure 3:**
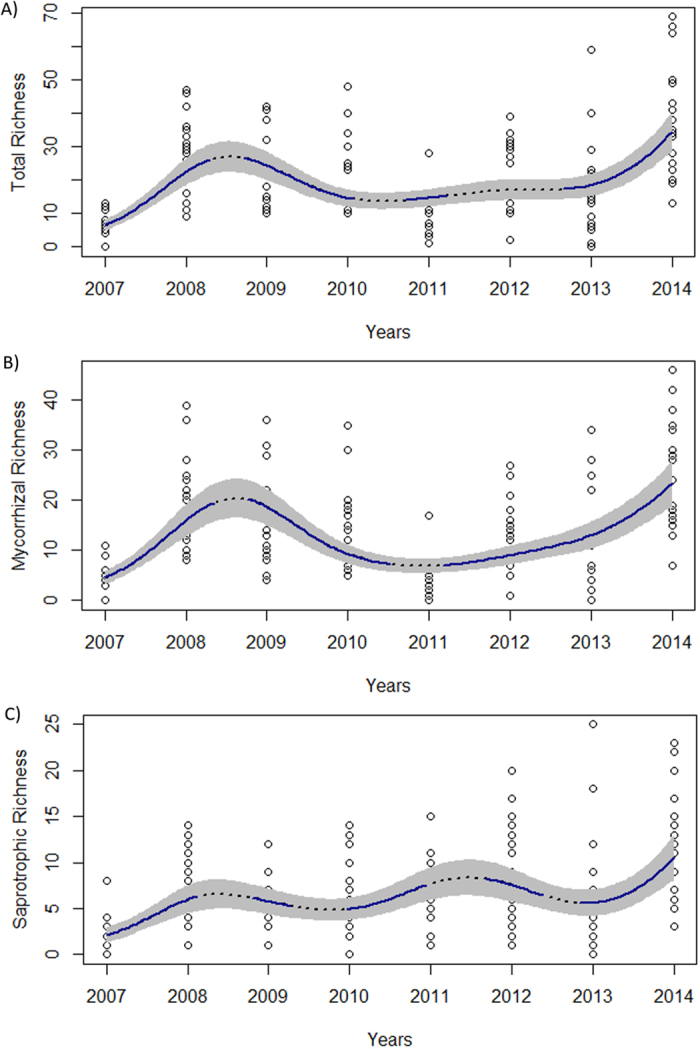
Temporal patterns fits of epigeous sporocarps richness in *P. sylvestris* forest stands along an elevational gradient within a Mediterranean region (Catalonia): (**A**) total richness, (**B**) mycorrhizal richness, (**C**) saprotrophic richness. Each point represent a sample plot. Continuous blue lines indicate significant temporal decreases or increases in richness (*P* < 0.05; GAMMs). Grey envelops are 95% confidence intervals.

The additive modelling demonstrated that diversity trends through time (total, mycorrhizal and saprotrophic) were non-linear (Fig. 3). Total and mycorrhizal richness showed similar trends through time, being characterized by a significant increase in 2008–2009 years up to 24 ± 2.5 and 19 ± 2.1 species per plot for total and mycorrhizal richness, respectively (hump-shape; Fig. 3A,B). This was followed by a significant reduction in 2010. Afterwards, total richness showed fairly constant values around 17 ± 1.9 species per plot (2010–2012), whereas mycorrhizal richness showed a minimum of 7 ± 0.9 species per plot in 2011. Then, both models showed a significant richness increase from 2013, with a maximum in 2014 of 34 ± 3.7 and 23 ± 2.8 species per plot, respectively. In contrast, saprotrophic richness showed a different trend, mainly characterized by three periods of significant richness increase: 2007–2008, 2010–2011 and 2013–2014 (Fig. 3C). Overall, saprotrophic richness increases from 2 ± 0.6 to 11 ± 1.4 species per plot over the eight-year period (2007–2014). There was a positive significant linear relationship between the three richness and biomass variables, being very important for total (R^2^ = 0.65) and mycorrhizal fungi (R^2^ = 0.70) and less important in the case of saprotrophic fungi (R^2^ = 0.44).

Surprisingly, total, mycorrhizal and saprotrophic richness variables showed no significant trend with elevation (*P* > 0.05; Table 2). However, analysis of elevational trends by years reveals a significant linear increasing trends through elevation for total and mycorrhizal richness in 2009 (slope_total_ = 0.29 ± 0.12, P = 0.017) and 2013 (steeper slope_total_ = 0.86 ± 0.15, *P* < 0.001), whereas saprotrophic richness increased only in 2013 (slope = 0.87 ± 0.20, *P* < 0.001). In relation with climatic drivers, the only positive significant relationship was found with LSEA precipitation (Table 2), indicating that epigeous sporocarps richness increases with increasing LSEA precipitation, regardless of the functional guild considered.

### Sporocarp evenness through time and elevation

Only saprotrophic evenness showed a significant linear trend with elevation (increasing, *P* < 0.05; Table 2). In the same way, the analysis of elevational trends by years revealed a lack of significant trends for total and mycorrhizal evenness (*P* > 0.05). In contrast, saprotrophic evenness showed a significant linear increasing trends with elevation in 2007 (slope_sapro_ = 0.17 ± 0.09, *P* = 0.050) and 2012 (slope_sapro_ = 0.11 ± 0.04, *P* = 0.033). Climatic analysis showed that the most important driver for total and mycorrhizal evenness was LSEA precipitation (Table 2), indicating that both variables increase with increasing LSEA precipitation. LSEA mean temperature was only positively related with mycorrhizal evenness (*P* < 0.05; Table 2), indicating a slightly increase with increasing LSEA mean temperature.

### Yearly fluctuations on biomass and diversity

**Figure 4:**
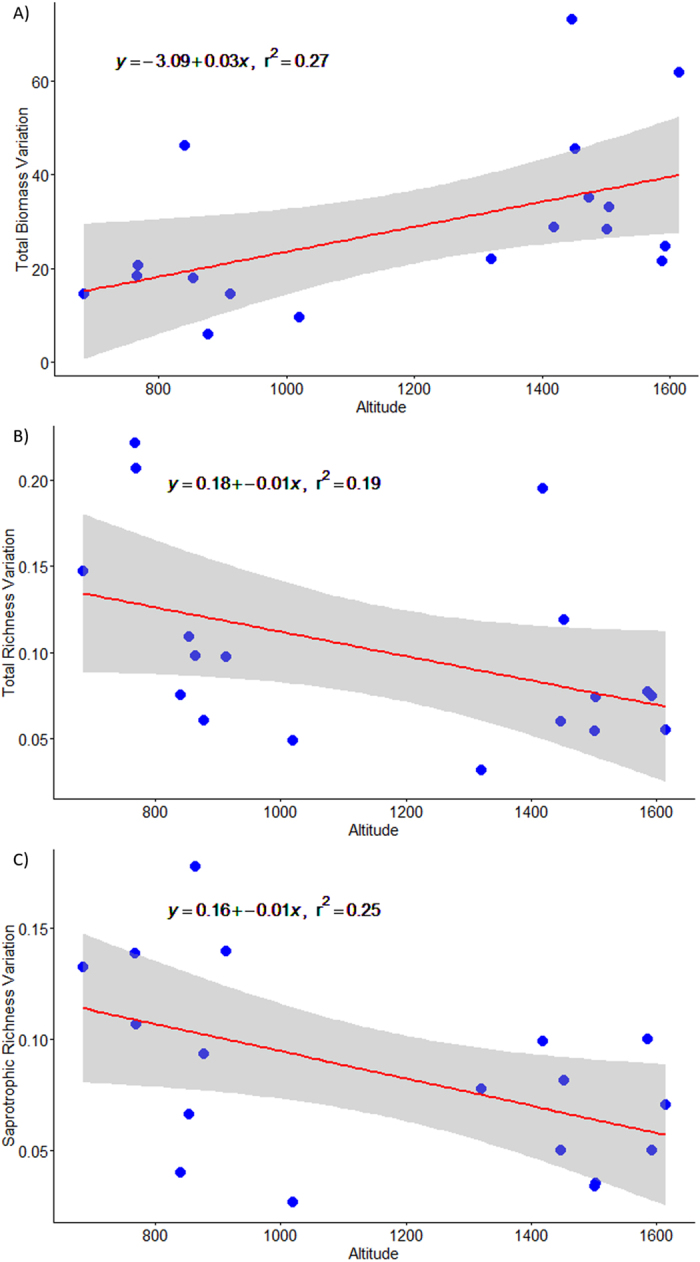
Yearly component of variation for each plot through elevation for epigeous sporocarps total biomass and richness in *P. sylvestris* forest stands along an elevational gradient within a Mediterranean region (Catalonia): (**A**) total biomass, (**B**) saprotrophic richness and (**C**) total richness. Altitude is measured in meters and each blue point represent a sample plot.

Only total and mycorrhizal biomass showed a significant linear increase in the yearly component of variation with elevation, being similar in both cases (slope_total[1,16]_ = 0.03 ± 0.01, *P* = 0.028, R^2^ = 0.27; slope_myco[1,16]_ = 0.03 ± 0.01, *P* = 0.026, R^2^ = 0.27; Fig. 4A). In contrast, saprotrophic biomass showed no significant trend (*P* > 0.05). Therefore, the yearly total and mycorrhizal biomass variation is greater at higher elevations than at lower elevations. When analysing sporocarp richness we only found a significant decreasing trend with elevation for saprotrophic fungi (slope_sapro[1,17]_ = −0.01 ± 0.001, *P* = 0.025, R^2^ = 0.26; Fig. 4B) and close significance for total richness (slope_total[1,17]_ = −0.01 ± 0.001, *P* = 0.072, R^2^ = 0.19; Fig. 4C). Mycorrhizal richness showed no significant trend (*P* > 0.05). The yearly saprotrophic and total richness variation was greater at lower elevations than at higher elevations. Finally, the three evenness variables showed no significant trend in the yearly component of variation with elevation (*P* > 0.05), suggesting that evenness fluctuations are constant along the elevational gradient.

## Discussion

Whilst some studies have reported epigeous mushroom abundance and diversity patterns along elevation gradients from various regions^23^,^24^,^25^, the ecological scales of variation and the climate dependence of these relationships has not been investigated, especially in the Mediterranean region. Here, our results show that the epigeous biomass is mainly dependent on inter-annual variations, whereas richness and evenness are more spatial-scale dependent. Surprisingly, the elevational effect was less important than expected, although, clear differences in temporal distribution of biomass and richness between ectomycorrhizal and saprotrophic guilds were found. The main driver of these variations was LSEA precipitation, which was more influential than LSEA mean temperature in this area. These results suggest that different scale processes (inter-annual variations vs. plot-scale) are important in determining biomass and diversity spatio-temporal patterns depending on the functional guilds considered. Moreover, the high spatial and temporal variability found in epigeous sporocarp biomass and diversity in this work highlights the difficulty of characterizing fungal communities and their drivers, emphasising the importance of having long-term datasets of multiple spatial points.

Surprisingly, our results showed that total ectomycorrhizal biomass was much more important than total saprotrophic biomass across the study period (94% *vs*. 6%). This might be caused by a combination of (1) greater ectomycorrhizal sporocarp number, i.e. nearly doubling the total saprotrophic sporocarps number over 8 years, and (2) greater biomass per sporocarp. Recent works comparing sporocarps have shown that ectomycorrhizal species have larger sporocarps than saprotrophs^33^. Indeed, only 21 out of the 100 most abundant species in biomass, listed in Supplementary Table S1, are saprotrophic ones (e.g. *Lycoperdon perlatum, Macrolepiota procera, Rhodocollybia butyracea*), and only 1 is included among the 25 most productive species (*Leucopaxillus gentianeus*). In contrast, the most productive ectomycorrhizal species had biomass values over 2 kg/ha/yr (e.g. *Lactarius deliciosus, Suillus variegatus, L. vellereus, Tricholoma fracticum, Russula sanguínea, R. torulosa, Craterellus lutescens, S. luteus, L. chrysorrheus* and *Tricholoma portentosum*). Ectomycorrhizal species are clearly able to obtain more resources to produce greater sporocarp biomass than saprotrophs in these stands.

The patterns in biomass, richness and evenness variance across spatio-temporal scales revealed three important results: (i) biomass and richness variances are opposite distributed across years and plots, (ii) variation in ectomycorrhizal and saprotrophic guilds differed across spatio-temporal scales, and (iii) variation in evenness appears controlled at the plot level. First, the opposing distributions of biomass and richness variance among spatio-temporal scales suggests that different scale processes are important in determining sporocarp biomass and richness values. For example, there was greater yearly variation in biomass compared to richness (42% vs. 28%), supporting our prediction that yearly climatic changes (precipitation here) would modify epigeous sporocarp biomass production in these Mediterranean *P. sylvestris* stands. Similar results have been found by De-la-Varga *et al*.^34^ and Ágreda *et al*.^20^ in Mediterranean Spain, and by Heegaard *et al*.^35^ in Switzerland. It seems that temporal effects, here arising from yearly environmental variations, should be taken into account in epigeous sporocarps biomass production studies^36^, because they will affect the results obtained and conclusions drawn^37^. In contrast to our expectations, richness showed greater within-space variation than temporal variation (64% spatial vs. 28% temporal, Table 1), which suggest that sporocarp richness is mainly dependent on common species compositional differences between localities and plots^38^.

Second, the different distribution of variance in biomass and richness within spatio-temporal scales between ectomycorrhizal and saprotrophic fungi is in agreement with previous studies^35^. Here, the greater fraction of variance for ectomycorrhizal fungi occurred between years (44% and 43% for biomass and richness, respectively), whereas for saprotrophic fungi the greater fraction occurred between plots (56% and 54% for biomass and richness, respectively). It is well known that ectomycorrhizal and saprotrophic fungi differ in methods for obtaining carbon and other nutrients which affects their fruiting^39^. Ectomycorrhizal fungi are root symbionts that rely on photosynthate carbon flux from host plants^16^. However, in these Mediterranean climates where *P. sylvestris* is at its most southerly distribution, tree growth is constrained by drought and high temperatures during summer^40^, the consequence of which brings about an increased leaf turnover^40^. Thus, in adverse years trees need to invest more carbon in maintaining canopy functioning, i.e. sustaining leaf biomass and its turnover^41^, and reducing the amounts available for the mycorrhizal fungi^42^. Therefore, yearly seasonal variations in host plants photosynthetic rates determine the available carbohydrates which are accessible for ectomycorrhizal fungal fruiting^39^. In contrast, saprotrophic fungi are decomposers obtaining carbon directly from organic matter (leaf litter and coarse woody debris)^43^, which are clearly reliant on plot-level processes involved in wood debris and leaf litter production. Thus, it seems that there are two main environmental filters operating at different scales for epigeous sporocarps biomass production and diversity, i.e. the year-scale for ectomycorrhizal and the plot-scale for saprotrophic fungi.

Third, we found that localities and years explained residual proportions of variance across epigeous sporocarp evenness (Table 1, Fig. 1), suggesting that these spatial and temporal factors do not affect variation in evenness. Thus, epigeous sporocarp evenness variation is conditioned by community-level processes at plot-scale. Clearly, between-plot variations (i.e. niche availability or resources limitations) have great consequences for sporocarp compositional dynamics, and hence for sporocarp evenness.

In general, non-linear trends through time in biomass and richness were found for total and ectomycorrhizal species. There were significant increases and decreases in both variables resulting in peaks in some years (2009 and 2014; Figs 2 and 3). Mainly LSEA precipitation and, to lesser extent, mean temperature were able to explain biomass and richness patterns. The relationships of precipitation and temperature with fruiting bodies has been described before within Mediterranean region^26^,^44^, although the inter-annual variations are generally ignored. Fungi need water to mature their reproductive structures consequently more individuals are found in years when LSEA precipitation are greater^45^.

Our results, based on plots with high variability, do not reveal the general decreasing trend in sporocarp biomass and richness for ectomycorrhizal fungi detected by Ágreda *et al*.^20^. Such decreasing and increasing trends might suggest the existence of pulses in biomass, and to a lesser extent in richness, as a response to the inter-annual irregularity in precipitation typical of Mediterranean areas^46^. Here, the study sites in some years had an extremely low productivity, with biomass less than 5 kg per hectare in unfavourable years (2007 and 2011; Fig. 2A). Therefore, it seems that large productivity is confined to those years when climatic conditions are more optimal. Thus, when late-summer and early-autumn precipitation and temperature are optimal, fungal species produce sporocarps in abundance^47^. Based on our results, this seems to be at least every 5–7 years. In any case, in our study areas the epigeous sporocarps patterns might be driven by occasional pulses (mainly arising from differences in LSEA precipitation). If true, then the increase in precipitation irregularity produced by global change might reduce the frequency of these pulses, i.e. increasing the time lag between pulses (increasing return time^48^). Although, we do not present any long-term temporal trend to validate this hypothesis, there is evidence for sustained low sporocarps biomass production over the last few decades in Mediterranean areas of Spain with exceptionally greater productivity in some years (e.g. 2014)^38^.

Saprotrophic fungi showed an increasing trend in biomass and richness patterns through time, which was somewhat unexpected. The recent reductions in forest management over the last 10 years^37^ combined with the increasing forest maturity may have resulted in the proliferation of litter, woody debris and dead trunks in these plots. As a consequence, niche and resource availability for saprotrophic species have increased^49^, and hence, their biomass and richness. Similar increases of saprophytic species with time have been reported in boreal regions^19^. However, our outcomes clearly reveal that saprotrophic increases are plot-scale dependent and driven by LSEA precipitation, which are in contrast to findings from boreal regions which report that saprotrophic plot-variations were not relevant^19^. Thus, in this Mediterranean area it seems that plot-scale niche processes are the main filters for saprotrophic sporocarp production and richness.

Surprisingly, the elevational analysis revealed only an increase in saprotrophic evenness; biomass, richness and other evenness variables showed no pattern with elevation. As mentioned above, LSEA precipitation drove saprotrophic production and diversity, furthermore, LSEA precipitation during the study period were greater at higher elevations (500–850 m = 1,107 mm vs. >1500 m = 1,660 mm cumulative values for the eight-year study period). Therefore, the existence of more equitability of saprotrophic species abundances at higher elevations might be related to greater water availability in late-summer and early-autumn. In contrast, in low elevations inter-specific competition between saprotrophic species for resources^50^, here water availability, might be responsible of the low saprotrophic diversity and yield.

A number of studies have found biomass and richness changes with elevation^23^,^26^,^30^. These studies have reported different type of responses (i.e. decreasing trends and mid-elevation peaks). However, here the temporal patterns in 8 out of the 9 variables analysed were independent of elevation. This result may be explained by the features of the Mediterranean elevational gradient under study compared to studies in temperate, boreal and tropical zones^23^,^24^,^25^. That is, in our 931 m elevational gradient, temperature seemed to be less important than LSEA precipitation for sporocarp biomass and richness (see Table 2). At the same time, spatial distribution irregularity of late-summer and early-autumn rainfalls is common in this area^51^. Thus, the inter-annual variation of precipitation (i.e. amount and distribution) in this Mediterranean area are more influent than the expected elevational effect associated with changes in temperature^51^. In contrast, in temperate and boreal gradients temperature appears to be the most limiting factor for fungal fruiting^23^,^24^. However, when yearly elevational changes were considered, significant increasing or decreasing trends for biomass were found in consecutive years (2012 and 2013), and increasing trends for richness (2009 and 2013). Such patterns are clear evidence of the importance of inter-annual variations in precipitation for determining biomass and diversity patterns for ectomycorrhizal and saprotrophic species in elevational gradients in the Mediterranean region. In any case, the inexistence of an elevational effect over the whole biomass production does not mean a lack of elevational effect when biomass is split in different genera or groups of species, as was demonstrated in previous works for edible mushrooms production in similar areas^37^. This suggests that increasing the accuracy in epigeous sporocarp yield modelling may require approaches that consider individual fungal species or groups with similar features (functional groups) instead of using the whole sporocarp community^47^.

The yearly detected biomass variances increased with elevation (total and mycorrhizal), being greater at higher elevations than at lower elevations; this was in line with our hypotheses. As the optimal climatic conditions for producing fruiting bodies are more frequent at higher elevations (greater LSEA rainfall), there is inherently more opportunity for variation at these sites. Surprisingly, at lower elevations sporocarp biomass variances were lower than expected; this might be produced by a combination of (i) an inherent lower productivity at low elevations, even in optimal years, and (ii) because precipitation during the study period was not enough to generate long-enough-lasting windows of opportunity for fungi to produce abundant epigeous sporocarps. Previous work at the lower part of the elevational gradient has demonstrated that *P. sylvestris* is located at its lower distributional edge^32^. These works predicted low ecosystem productivities limited mainly by a lack of water and high temperatures, which might also be related to the lower sporocarp yields and variability reported here (mean values were less than 5 kg per ha in lower elevational areas). These results have some interesting implications; when the climatic conditions in LSEA are not optimal, mainly produced by a lack of precipitation, there is low sporocarp productivity; whereas when water resource availability is more favourable there will be a pulse of production. Similar pulses of production have been reported for *Pinus halepensis* stands when conditions are favourable in Mediterranean Spain^52^.

In contrast to biomass, sporocarp richness (saprotrophic and partially-total) showed decreasing variances with elevation, indicating greater variations in the number of saprotrophic species at lower compared to higher elevations. These results, being opposite to biomass, are also driven by a combination of low productivity and precipitation at the low gradient edge. Here, the unfavourable conditions during study period produced greater yearly variations in sporocarp richness at low elevation compared to higher elevations, where inter-annual climatic conditions, being more optimal, are also more favourable even in the worst years than in lower elevational plots. Finally, there was a constancy of evenness fluctuations along the elevational gradient, indicating that spatial processes are more important than inter-annual variations in determining sporocarp evenness patterns.

The evidence reported here suggests that epigeous sporocarp fruiting patterns vary between ecological scales, with biomass mainly dependent on inter-annual variations (i.e. yearly changes in environmental conditions), whereas richness and evenness are more spatial-scale dependent. Temporal effects influences patterns of biomass and diversity strongly, being different even between ectomycorrhizal and saprotrophic species. These inter-annual variations are largely explained by variation in late-summer and early-autumn precipitation rather by mean temperature. The ubiquitous role of precipitation across fungi guilds and spatio-temporal scales implies that it is a limiting resource (general mechanism) controlling sporocarp production and diversity in this Mediterranean region.

## Materials and Methods

### Study area

Mushrooms were measured in Scots Pine (*P. sylvestris*) forest stands located along a south to north gradient running through Catalonia region of north-eastern Spain (Lat: 42°28′18.1236″ to 41°19′51.38″N; Long: 1°21′15.192″ to 1°2′51.76″W). The climate is Mediterranean although there is considerable variation from south to north in accordance with the elevational gradient (almost 1,000 m). Mean annual rainfall between 2007 and 2014 along the gradient ranged from 595 to 1,051 mm (increasing with elevation); with an intense summer drought period from June to August. Mean annual temperatures tend to decline with increasing elevation and ranged from 9° to 6.2 °C. Soils are sandy-loam or loam, whereas vegetation is dominated by Scots pine, *Buxus sempervirens* and *Quercus* spp.

### Sampling design

Sampling started in 2007 with the establishment of 14 plots (10 × 10 m) randomly selected in *P. sylvestris* stands located at different elevations (elevation interval: 850–1150 m 4 plots, 1150–1500 m 5 plots, and >1500 m 5 plots). In 2008, 5 new plots were established in the southern part of the gradient to capture the lower elevational information (elevation interval: 500–850 m 4 plots and 1 new plot in 850–1150 m interval). The selected plots can be grouped in four localities (Pyrenees 1,604 m; pre-Pyrenees 1,422 m; Central-depression 1,180 m and Coast-range 785 m) according to differences in the climatic conditions. Thus, our dataset comprises 19 permanent plots ranging from 684 to 1,615 m.a.s.l., all sampled in eight consecutive years (2007–2014), making it the largest standardized dataset considering both spatial and temporal effects on epigeous sporocarps available over Scots pine forest elevational gradient in Mediterranean climate. All the selected *P. sylvestris* stands were mono-specific naturalized forests that have not been managed in the recent years, as a consequence of the widespread abandonment of forest management in Catalonia^37^. However, the type of management over a longer historical period has been comparable in all stands; i.e. selective cuttings aiming at harvesting the most valuable timber only, which has resulted in similar stand structure^37^. For each elevation interval, there is indeed slight variability in terms of the stand density between plots (tree density).

### Data collection

Plots were sampled weekly from the beginning of September to the end of December each year between 2007 and 2014. Each week, all epigeous sporocarps were counted and harvested from each of the 19 plots. In the laboratory, collected sporocarps were identified using morphological features and then their fresh-weight determined. Identification was to species level where possible, otherwise to genus or subgenus level. Simultaneously, species were classified considering their trophic strategy; i.e. mycorrhizal or saprotrophic, according to the existing literature and expert knowledge^15^. Climatic data were obtained from the nearest meteorological stations (i.e. mean distance around 5 km from the plots). Here, we only considered the August to October accumulated precipitation and mean temperature to characterize the main seasonal drivers of “late-summer and early-autumn” (LSEA) epigeous sporocarps fruiting. The mushroom biomass (kg fresh weight ha^−1^) used in the analysis are total values recorded per plot and year^53^. Species richness (S) is the number of different species per plot and year, and Pielou’s evenness index (J) was obtained using annual biomass values of different species^54^.

### Data analysis

All statistical analyses were performed in R statistical environment (3.2.2R Core Team, 2015, The R Foundation for Statistical Computing, Vienna, Austria). Variance component analyses for biomass and diversity were performed using “ape”^55^ and “nlme” packages^56^; while Generalized Additive Mixed Models were fitted using the “mgcv” package (GAMM^57^).

Here, we used three statistical approaches to provide an overview of sporocarps productivity and diversity trends and variations at different spatio-temporal scales including elevation. First, we started identifying ecological scales (spatial and temporal) that account for a large percentage of total variance around the mean in biomass (kg ha^−1^) and diversity (richness and evenness); these variables were also analysed by functional guilds. We performed a variance component analysis over a linear mixed model (*“varcomp”* function), using a restricted maximum likelihood method (REML). Here, the ecological scales considered were: plot, locality and year. Therefore, a null model was fitted including plots nested within localities nested within years as random effects. The 95% confidence intervals of variance components were calculated using bootstrapping techniques (199 runs with replacement). Biomass and diversity variables were normalized using square-root and log-transformations, respectively. Identification of the spatio-temporal scales that account for most of the variation in biomass and diversity is a first step to describe the most important sporocarp patterns and processes accurately^58^.

Second, three different GAMMs were used to examine the extent to which sporocarps biomass, richness and evenness (both split in functional guilds) vary through time and elevation, and how they are related with late-summer and early-autumn precipitation and mean temperature. This approach was chosen because it makes no *a priori* assumption about the functional relationship between variables. (i) The first model describe only the main temporal patterns of response variables (biomass, richness and evenness), here only time (in years) was used as explanatory variable. (ii) The second model examine the elevational variation of response variables and the extent to which these variables are related to LSEA precipitation and mean temperature. Here, elevation, LSEA precipitation and mean temperature were included as explanatory variables. Finally, (iii) the third model examine the yearly elevational changes of response variables. In all three models a Poisson error distribution was used for sporocarp richness variables, while biomass and evenness were square-root and arcsine transformed, respectively, and in both sets of models a Gaussian error structure was used^59^. In all models, plots nested within localities were included as random factors to account for spatial autocorrelation. Simultaneously, we accounted for temporal autocorrelation in the residuals by adding a first-order autoregressive process structure^55^.

We identify the periods where biomass and diversity were either increasing or decreasing in a significant way inside the GAMM models as tentative evidence for cycling or pulses^60^. The first derivative of the best GAMM models fit line was computed using the finite differences method^60^. A new time vector of 100 equally spaced time points over the observation period was used to obtain the fitted values from best-fits. Then, this time vector was slightly shifted in time and the fitted values recalculated, therefore, the differences between the two sets of fitted values, divided by the difference in time, yield the first derivatives of the trend^60^. We also computed standard errors and a 95% point-wise confidence interval for the first derivative. The trend was subsequently deemed significant when the derivative confidence interval was bounded away from zero at the 95% level (for full details on this method see ref. 60).

Third, to test the hypothesis that the biomass and diversity fluctuations will be greater in the lower and upper extremes of the elevational gradient far from optimal, we calculated the yearly component of variation per each plot. Here, we extracted from each plot-time series the yearly variances for each variable (biomass, richness and evenness) using generalized linear mixed models (*“varcorr”* function), considering years as random-effects terms and under REML. Finally, we regressed these values against elevation using linear models to identify whether significant greater variations were produced in the lower and upper extremes of the elevational gradient.

## Additional Information

**How to cite this article:** Alday, J. G. *et al*. Mushroom biomass and diversity are driven by different spatio-temporal scales along Mediterranean elevation gradients. *Sci. Rep.* **7**, 45824; doi: 10.1038/srep45824 (2017).

**Publisher's note:** Springer Nature remains neutral with regard to jurisdictional claims in published maps and institutional affiliations.

**Publisher's note:** Springer Nature remains neutral with regard to jurisdictional claims in published maps and institutional affiliations.

## Supplementary Material

Supplementary Table S1

## Figures and Tables

**Table 1:** Percentage of variance for total biomass and diversity variables across three ecological scales (plot, locality and year).

Ecological scales	Error	Plot	Locality	Year
**Variables**				
Total biomass	7 [6.3–7.1]	34 [31–37]	17 [13–21]	42 [40–45]
Total richness	7 [6.6–7.3]	35 [31–37]	29 [25–34]	28 [22–33]
Total evenness	6.5 [6–8]	86 [78–93]	7 [0–15]	0.5 [0–4]

Bootstrapped 95% confidence intervals are represented inside square brackets.

**Table 2:** Fixed parameters estimates from GAMMs describing temporal and elevational changes in sporocarp biomass and diversity and their relationships with LSEA precipitation (mm) and temperature (°C) in *P. sylvestris* forest stands along an elevational gradient within a Mediterranean region (Catalonia).

Fixed terms	Year	Elevation	Precipitation	Temperature
**Dependent variables**				
Total biomass	**14**.**92** **±** **2**.85***	0.03 ± 0.65	**6**.**74** **±** **2**.80**	**1**.**52** **±** **0**.65**
Mycorrhizal biomass	**14**.**22** **±** **2**.89***	0.12 ± 0.63	**6**.**17** **±** **2**.88*	**1**.**60** **±** **0**.54**
Saprotrophic biomass	**0**.**37** **±** **0**.11***	−0.19 ± 0.27	**2**.**62** **±** **1**.10*	0.43 ± 0.39
Total richness	**2**.**17** **±** **0**.40***	0.05 ± 0.08	**1**.**29** **±** **0**.44**	0.10 ± 0.12
Mycorrhizal richness	**2**.**11** **±** **0**.41***	0.02 ± 0.07	**1**.**55** **±** **0**.60**	0.07 ± 0.07
Saprotrophic richness	**2**.**18** **±** **0**.44***	0.02 ± 0.11	**2**.**31** **±** **0**.74**	−0.04 ± 0.25
Total evenness	−0.01 ± 0.01	0.02 ± 0.02	**0**.**18** **±** **0**.08*	−0.02 ± 0.03
Mycorrhizal evenness	−0.01 ± 0.01	0.01 ± 0.02	**0**.**12** **±** **0**.07*	**−0**.**05** **±** **0**.02*
Saprotrophic evenness	0.01 ± 0.02	**0**.**05** **±** **0**.02*	−0.03 ± 0.02	−0.02 ± 0.02

The dependent variables were also split by mycorrhizal and saprotrophic guilds. Estimates (±SE) are presented along with significance of each term. Significant parameters are represented in bold: *P < 0.05; **P < 0.01; ***P < 0.001.

## References

[b1] Körner C. The use of ‘altitude’ in ecological research. Trends Ecol. Evol. 22, 569–574 (2007). 17988759 10.1016/j.tree.2007.09.006

[b2] Solomon S. (Ed.) Climate change 2007-the physical science basis in Working group I contribution to the fourth assessment report of the IPCC(Vol. 4) (Cambridge University Press, 2007).

[b3] Morueta-Holme N. . Strong upslope shifts in Chimborazo’s vegetation over two centuries since Humboldt. Proc. Natl. Acad. Sci. USA 112, 12741–12745 (2015). 26371298 10.1073/pnas.1509938112 PMC4611603

[b4] Tripp E. A. . Biodiversity gradients in obligate symbiotic organisms: exploring the diversity and traits of lichen propagules across the United States. J. Biogeogr., doi: 10.1111/jbi.12746 (2016).

[b5] Büntgen U. . Truffles and climate change. Front. Ecol. Environ. 9(3), 150–151 (2011).

[b6] Büntgen U. . Drought-induced changes in the phenology, productivity and diversity of Spanish fungi. Fungal Ecol. 16, 6–18 (2015).

[b7] Andrew C. J., Van Diepen L. T., Miller R. M. & Lilleskov E. A. Aspen-associated mycorrhizal fungal production and respiration as a function of changing CO 2, O 3 and climatic variables. Fungal Ecol. 10, 70–80 (2014).

[b8] Andrew C. . Climate impacts on fungal community and trait dynamics. Fungal Ecol. 22, 17–25 (2016).

[b9] Dighton J., White J. F. Jr., White J. & Oudemans P. ed. The fungal community: its organization and role in the ecosystem(CRC Press, 2005).

[b10] Bebber D. P., Ramotowski M. A. & Gurr S. J. Crop pests and pathogens move polewards in a warming world. Nat. Clim. Change. 3(11), 985–988 (2013).

[b11] Bebber D. P., Holmes T. & Gurr S. J. The global spread of crop pests and pathogens. Glob. Ecol. Biogeogr. 23(12), 1398–1407 (2014).

[b12] Alday J. G. . Record breaking mushroom yields in Spain. Fungal Ecol. doi: 10.1016/j.funeco.2017.01.004 (2017).

[b13] Kauserud H. . Warming-induced shift in European mushroom fruiting phenology. Proc. Natl. Acad. Sci. USA 109(36), 14488–14493 (2012). 22908273 10.1073/pnas.1200789109 PMC3437857

[b14] Gehring C. A., Theimer T. C., Whitham T. G. & Keim P. Ectomycorrhizal fungal community structure of pinyon pines growing in two environmental extremes. Ecology 79(5), 1562–1572 (1998).

[b15] Tedersoo L. . Global diversity and geography of soil fungi. Science 346(6213), p.1256688 (2014). 10.1126/science.1256688 25430773

[b16] Smith S. E. & Read D. J. Mycorrhizal Symbiosis(Academic Press, 2008).

[b17] Eastwood D. C. . The plant cell wall–decomposing machinery underlies the functional diversity of forest fungi. Science 333, 762–765 (2011). 21764756 10.1126/science.1205411

[b18] Clemmensen K. E. . Roots and associated fungi drive long-term carbon sequestration in boreal forest. Science 339, 1615–1618 (2013). 23539604 10.1126/science.1231923

[b19] Straatsma G. . Species richness, abundance, and phenology of fungal fruit bodies over 21 years in a Swiss forest plot. Mycol. Res. 105, 515–523 (2001).

[b20] Ágreda T. . Increased evapotranspiration demand in a Mediterranean climate might cause a decline in fungal yields under global warming. Global Change Biol. 21, 3499–3510 (2015). 10.1111/gcb.12960 25930066

[b21] Büntgen U. . Linking climate variability to mushroom productivity and phenology. Front. Ecol. Environ. 10, 14–19 (2011).

[b22] Pickles B. J., Egger K. N., Massicotte H. B. & Green D. S. Ectomycorrhizas and climate change. Fungal Ecol. 5(1), 73–84 (2012).

[b23] Nouhra E. R. . Differential hypogeous sporocarp production from *Nothofagus dombeyi* and *N. pumilio* forests in southern Argentina. Mycologia 104, 45–52 (2012). 21914828 10.3852/11-098

[b24] Vishwakarma M. P. . Macrofungal diversity in moist temperate forests of Garhwal Himalaya. Indian J. Sci. Technol. 5, 1928–1932 (2012).

[b25] Gómez-Hernández M. & Williams-Linera G. Diversity of macromycetes determined by tree species, vegetation structure, and microenvironment in tropical cloud forests in Veracruz. Botany 89, 203–216 (2011).

[b26] Rincón A. . Compartmentalized and contrasted response of ectomycorrhizal and soil fungal communities of Scots pine forests along elevation gradients in France and Spain. Environ. Microbiol. 17, 3009–3024 (2015). 25953485 10.1111/1462-2920.12894

[b27] Nogués-Bravo D. N., Araújo M. B., Lasanta T. & Moreno J. I. L. Climate change in Mediterranean mountains during the 21st century. AMBIO. 37(4), 280–285 (2008). 18686507 10.1579/0044-7447(2008)37[280:ccimmd]2.0.co;2

[b28] Matías L. & Jump A. S. Asymmetric changes of growth and reproductive investment herald altitudinal and latitudinal range shifts of two woody species. Global Change Biol. 21, 882–896 (2015). 10.1111/gcb.12683 25044677

[b29] Scheffer M. . Anticipating critical transitions. Science 338, 344–348 (2012). 23087241 10.1126/science.1225244

[b30] Miyamoto Y. . The mid-domain effect in ectomycorrhizal fungi: range overlap along an elevation gradient on Mount Fuji, Japan. ISME J. 8, 1739–1746 (2014). 24621523 10.1038/ismej.2014.34 PMC4817612

[b31] Colwell R. K. . The mid‐domain effect and species richness patterns: What have we learned so far? Am. Nat. 163, 1–23 (2004). 15026983 10.1086/382056

[b32] Vilà-Cabrera A. . Structural and climatic determinants of demographic rates of Scots pine forests across the Iberian Peninsula. Ecol. Appl. 21, 1162–1172 (2011). 21774421 10.1890/10-0647.1

[b33] Bässler C., Heilmann-Clausen J., Karasch P., Brandl R. & Halbwachs H. Ectomycorrhizal fungi have larger fruit bodies than saprotrophic fungi. Fungal Ecol. 17, 205–212 (2015).

[b34] De-la-Varga H. . Seasonal dynamics of *Boletus edulis* and *Lactarius deliciosus* extraradical mycelium in pine forests of central Spain. Mycorrhiza 23, 391–402 (2013). 23392533 10.1007/s00572-013-0481-3

[b35] Heegaard E. . Fine‐scale spatiotemporal dynamics of fungal fruiting: prevalence, amplitude, range and continuity. Ecography, doi: 10.1111/ecog.02256 (2016).

[b36] Boddy L. . Climate variation effects on fungal fruiting. Fungal Ecol. 10, 20–33 (2014).

[b37] de-Miguel S. . Impact of forest management intensity on landscape-level mushroom productivity: a regional model-based scenario analysis. For. Ecol. Manage. 330, 218–227 (2014).

[b38] Alday J. G. . Overcoming resistance and resilience of an invaded community is necessary for effective restoration: a multi‐site bracken control study. J. Appl. Ecol. 50, 156–167 (2013).

[b39] Sato H. . A thirty-year survey reveals that ecosystem function of fungi predicts phenology of mushroom fruiting. PloS one 7, e49777 (2012). 23209598 10.1371/journal.pone.0049777 PMC3507881

[b40] Sabaté S., Gracia C. A. & Sánchez A. Likely effects of climate change on growth of *Quercus ilex, Pinus halepensis, Pinus pinaster, Pinus sylvestris* and *Fagus sylvatica* forests in the Mediterranean region. For. Ecol. Manag. 162(1), 23–37 (2002).

[b41] Camarero J. J., Guerrero-Campo J. & Gutiérrez E. Tree-ring growth and structure of *Pinus uncinata* and *Pinus sylvestris* in the Central Spanish Pyrenees. Arct. Alp. Res. 1–10 (1998).

[b42] Kiers E. T. . Reciprocal rewards stabilize cooperation in the mycorrhizal symbiosis. Science 333, 880–882 (2011). 21836016 10.1126/science.1208473

[b43] Lindahl B. D. . Spatial separation of litter decomposition and mycorrhizal nitrogen uptake in a boreal forest. New Phytol. 173, 611–620 (2007). 17244056 10.1111/j.1469-8137.2006.01936.x

[b44] Bonet J. A. . Modelling the production and species richness of wild mushrooms in pine forests of the Central Pyrenees in northeastern Spain. Can. J. For. Res. 40, 347–356 (2010).

[b45] Martínez-Peña F. . Yield models for ectomycorrhizal mushrooms in *Pinus sylvestris* forests with special focus on *Boletus edulis* and *Lactarius* group *deliciosus*. For. Ecol. Manage. 282, 63–69 (2012).

[b46] Inglima I. . Precipitation pulses enhance respiration of Mediterranean ecosystems: the balance between organic and inorganic components of increased soil CO2 efflux. Global Change Biol. 15, 1289–1301 (2009).

[b47] Ágreda T., Águeda B., Fernández-Toirán M., Vicente-Serrano S. M. & Òlano J. M. Long-term monitoring reveals a highly structured interspecific variability in climatic control of sporocarp production. Agr. Forest. Meteorol. 223, 39–47 (2016).

[b48] Yang L. H. . What can we learn from resource pulses. Ecology 89, 621–634 (2008). 18459327 10.1890/07-0175.1

[b49] Buée M. . Soil niche effect on species diversity and catabolic activities in an ectomycorrhizal fungal community. Soil Biol. Biochem. 39, 1947–1955 (2007).

[b50] Tiunov A. V. & Scheu S. Facilitative interactions rather than resource partitioning drive diversity‐functioning relationships in laboratory fungal communities. Ecol. Lett. 8, 618–625 (2005).

[b51] Cortesi N. . Spatial variability of precipitation in Spain. Reg. Environ. Change 14, 1743–1749 (2014).

[b52] Martínez-de-Aragón J. . Manual de buenas prácticas para la gestión del recurso micológico forestal(CTFC, 2012).

[b53] Bonet J. A. . The relationship between forest age and aspect on the production of sporocarps of ectomycorrhizal fungi in *Pinus sylvestris* forests of the central Pyrenees. For. Ecol. Manage. 203, 157–175 (2004).

[b54] Magurran A. E. Measuring biological diversity(Blackwell Publishing, 2004).

[b55] Paradis E. . APE: analyses of phylogenetics and evolution in R language. Bioinformatics 20, 289–290 (2004). 14734327 10.1093/bioinformatics/btg412

[b56] Pinheiro J. . nlme: linear and nonlinear mixed effects models. R package version 3.1–122, URL: http://CRAN.R-project.org/package=nlme (2015).

[b57] Wood S. N. Fast stable restricted maximum likelihood and marginal likelihood estimation of semiparametric generalized linear models. J. Royal Stat. Soc. (B) 73, 3–36 (2011).

[b58] Messier J. . How do traits vary across ecological scales? A case for trait‐based ecology. Ecol. Lett. 13, 838–848 (2010). 20482582 10.1111/j.1461-0248.2010.01476.x

[b59] Crawley M. J. The R book(John Wiley & Sons, 2012).

[b60] Curtis C. J. & Simpson G. L. Trends in bulk deposition of acidity in the UK, 1988–2007, assessed using additive models. Ecol. Indic. 37, 274–286 (2014).

