# Supplementary material for: Mushroom biomass and diversity are driven by different spatio-temporal scales along Mediterranean elevation gradients
Source: Sci Rep. 2017 Apr 6;7:45824. doi: 10.1038/srep45824 (PMC5382911; doi:10.1038/srep45824)
Supplement: Supplementary Table S1 [file srep45824-s1.doc]

**Supplementary Information**

Mushroom biomass and diversity are driven by different spatio-temporal scales along Mediterranean elevation gradients

Josu G. Alday^1^; Juan Martínez de Aragón^2,3^ Sergio de-Miguel^1^, José Antonio Bonet^1,2^

^1^ Departament de Producció Vegetal i Ciència Forestal, Universitat de Lleida-Agrotecnio Center (UdL-Agrotecnio), Avda. Rovira Roure, 191, E-25198 Lleida, Spain

^2^ Centre Tecnològic Forestal de Catalunya (CTFC-CEMFOR). Ctra. de St. Llorenç de Morunys km 2, E-25280 Solsona, Spain

^3^ Forest Bioengineering Solutions S.A. Crta. de St. Llorenç de Morunys, Km. 2. E-25280 Solsona, Spain

Correspondence author: Josu G Alday

e-mail: [jgalday@pvcf.udl.cat](mailto:jgalday@pvcf.udl.cat%0Djosucham@gmail.com)

[josucham@gmail.com](mailto:jgalday@pvcf.udl.cat%0Djosucham@gmail.com)

Table S1: List of the 100 most productive species that have been identified during the monitoring period in *Pinus sylvestris* forest stands along an elevational gradient within a Mediterranean region (Catalonia). For each fungi are reported: species scientific name, mean biomass of epigeous sporocarps collected (kg/ha/yr) and their trophic strategy (M=mycorrhizal and S=saprotrophic).

| **Species list (Index fungarium 9-nov-2016)** | **Mean Biomass**  **(kg/ha/yr)** | **Trophic strategy** |
| --- | --- | --- |
| *Lactarius deliciosus* (L.) S. F. Gray | 8.41 | M |
| *Suillus variegatus* (Swartz) Rich. & Roze. | 5.36 | M |
| *Lactarius vellereus* (Fr.) Fr. | 4.64 | M |
| *Tricholoma fracticum* (Batsch) Kreisel | 3.37 | M |
| *Russula sanguinea* (Bull. ex St. Amans) Fr. | 2.99 | M |
| *Russula torulosa* Bres. | 2.88 | M |
| *Craterellus lutescens* (Fr.) Fr. | 2.61 | M |
| *Suillus luteus* (L.) Roussel. | 2.59 | M |
| *Lactarius chrysorrheus* Fr. | 2.21 | M |
| *Tricholoma portentosum* (Fr.) Quél. | 2.07 | M |
| *Hydnum repandum* L. | 2.00 | M |
| *Russula chloroides* (Kromb.) Bres. | 1.93 | M |
| *Hebeloma sinapizans* (Paul.) Gill. | 1.42 | M |
| *Hydnellum ferrugineum* (Fr.:Fr.) P. Karst. | 1.31 | M |
| *Hygrophorus latitabundus* Britz. | 1.25 | M |
| *Leucopaxillus gentianeus* (Quél.) Kotl. | 1.16 | S |
| *Lactarius sanguifluus* (Paul.) Fr. | 1.12 | M |
| *Chroogomphus rutilus* (Sch.) Miller | 1.03 | M |
| *Tricholoma terreum* (Sch.) Kumm. | 0.98 | M |
| *Russula albonigra* (Krombh.) Fr. | 0.96 | M |
| *Tricholoma virgatum* (Fr.) Kumm. | 0.88 | M |
| *Russula delica* Fr. | 0.86 | M |
| *Phellodon niger* (Fr.) P. Karst. | 0.85 | M |
| *Hebeloma laterinum* (Batsch) Vesterh. | 0.77 | M |
| *Tricholoma imbricatum* (Fr.) Kumm. | 0.67 | M |
| *Mycena zephirus* (Fr.) Kumm. | 0.62 | S |
| *Ramaria aurea* (Sch.) Quél. | 0.61 | M |
| *Suillus collinitus* (Fr.) Kuntze | 0.56 | M |
| *Lycoperdon perlatum* Pers. | 0.55 | S |
| *Boletus edulis* Bull. | 0.52 | M |
| *Sarcodon imbricatum* (L. ex Fr.) Karst. | 0.51 | M |
| *Macrolepiota procera* (Scop.) Sing. | 0.48 | S |
| *Cortinarius elegantior* (Fr.) Fr. | 0.47 | M |
| *Lactarius violascens* (Otto) Fr. | 0.42 | M |
| *Boletus pinophilus* Pil. & Derm. | 0.40 | M |
| *Cortinarius caninus* (Fr.) Fr. | 0.40 | M |
| *Russula badia* Quél. | 0.32 | M |
| *Russula paludosa* Britz. | 0.31 | M |
| *Suillus granulatus* (L.) Roussel. | 0.31 | M |
| *Rhodocollybia butyracea* (Bull.) Kumm. | 0.30 | S |
| *Cortinarius infractus* (Pers.) Fr. | 0.27 | M |
| *Scutiger pes-caprae* (Pers.) Bondartsev & Singer | 0.26 | M |
| *Suillus bovinus* (L.) Roussel | 0.26 | M |
| *Russula nigricans* Fr. | 0.25 | M |
| *Albatrellus ovinus* (Schaeff.) Kotl. & Pouzar | 0.24 | M |
| *Hebeloma crustuliniforme* (Bull. ex St. Amans) Quél. | 0.23 | M |
| *Rhizopogon luteolus* Fr. | 0.23 | M |
| *Russula integra* (L.) Fr. | 0.23 | M |
| *Inocybe terrigena* (Fr.) Kühn | 0.21 | M |
| *Lactarius* deliciosus (L.) S. F. Gray *x Hypomyces lateritius* | 0.21 | M |
| *Russula queletii* Fr. | 0.21 | M |
| *Clitocybe nebularis* (Batsch. ex Fr.) Kumm. | 0.20 | S |
| *Cortinarius amoenolens* Henry ex Orton | 0.20 | M |
| *Cortinarius decipiens* (Pers.) Fr. | 0.20 | M |
| *Leucopaxillus giganteus* (Leyss.) Sing. | 0.20 | S |
| *Amanita pantherina* (DC.) Krombh | 0.18 | M |
| *Gymnopilus penetrans* (Fr.) Murr. | 0.18 | S |
| *Inocybe glabripes* Ricken | 0.18 | M |
| *Russula sardonia* Fr. | 0.18 | M |
| *Clitocybe gibba* (Pers.) Kumm. | 0.17 | S |
| *Lactarius vinosus* Quél | 0.17 | M |
| *Lepiota magnispora* Murrill | 0.17 | S |
| *Pluteus cervinus* (Sch.) ex Kumm. | 0.17 | S |
| *Cortinarius fulmineus* (Fr.) Fr. | 0.16 | M |
| *Russula aurea* Pers. | 0.16 | M |
| *Russula heterophylla* (Fr.) Fr. | 0.16 | M |
| *Cortinarius calochrous* (Pers.) Fr. | 0.15 | M |
| *Mycena pura* (Pers.) Kumm. | 0.15 | S |
| *Pholiota gummosa* (Lasch) Sing. | 0.15 | S |
| *Amanita echinocephala* (Vitt.) Quél. | 0.14 | M |
| *Cortinarius evernius* (Fr.) Fr. | 0.14 | M |
| *Pholiota lenta* (Pers.) Sing. | 0.14 | S |
| *Rhizopogon roseolus* (Corda) Th. Fr. | 0.14 | M |
| *Tricholoma fulvum* (Bull.: Fr.) Sacc. | 0.14 | M |
| *Amanita ovoidea* (Bull.) Link. | 0.13 | M |
| *Mycena seynesii* Quél. | 0.13 | S |
| *Mycena leptocephala* (Pers.) Gill. | 0.12 | S |
| *Cortinarius subfulgens* Orton | 0.11 | M |
| *Leccinum versipelle* (Fr.) Snell | 0.11 | M |
| *Rhodocollybia maculata* (A.-S.) Kumm. | 0.11 | S |
| *Amanita muscaria* (L.) Hook. | 0.10 | M |
| *Chalciporus pierrhuguesii* (Boud). Bat | 0.10 | M |
| *Cortinarius privignus* (Fr.) Fr. | 0.10 | M |
| *Mycena galericulata* (Scop.) S.F. Gray. | 0.10 | S |
| *Ramaria flava* (Fr.) Quél. | 0.10 | M |
| *Russula adusta* (Pers.) Fr. | 0.10 | M |
| *Russula atropurpurea* Krombh. | 0.10 | M |
| *Russula densifolia* Gill. | 0.10 | M |
| *Russula fragilis* (Pers.) Fr. | 0.10 | M |
| *Collybia dryophila* (Bull.: Fr.) Kumm | 0.09 | S |
| *Cortinarius bulbosus* Fr. s. Rick | 0.09 | M |
| *Cortinarius cinnamomeus* (L.) Fr. | 0.09 | M |
| *Inocybe phaeodisca* Kühn. | 0.09 | M |
| *Russula alutacea* (Pers.) Fr. | 0.09 | M |
| *Russula luteotacta* Rea | 0.09 | M |
| *Russula turci* Bres. | 0.09 | M |
| *Chroogomphus helveticus* (Sing.) Moser. | 0.08 | M |
| *Clitocybe hydrogramma* (Bull. ex Fr.) Kumm. | 0.08 | S |
| *Paralepista flaccida* (Sowerby) Vizzini | 0.08 | S |
| *Russula caerulea* Fr. | 0.08 | M |
